# Noisy galvanic vestibular stimulation induces a sustained improvement in body balance in elderly adults

**DOI:** 10.1038/srep37575

**Published:** 2016-11-21

**Authors:** Chisato Fujimoto, Yoshiharu Yamamoto, Teru Kamogashira, Makoto Kinoshita, Naoya Egami, Yukari Uemura, Fumiharu Togo, Tatsuya Yamasoba, Shinichi Iwasaki

**Affiliations:** 1 Department of Otolaryngology, Faculty of Medicine, The University of Tokyo, 7-3-1 Hongo, Bunkyo-ku, Tokyo 113-8655, Japan; 2 Educational Physiology Laboratory, Graduate School of Education, The University of Tokyo, 7-3-1 Hongo, Bunkyo-ku, Tokyo 113-0033, Japan; 3 Biostatistics Division, Clinical Research Support Center, The University of Tokyo Hospital, 7-3-1 Hongo, Bunkyo-ku, Tokyo 113-8655, Japan.

## Abstract

Vestibular dysfunction causes postural instability, which is prevalent in the elderly. We previously showed that an imperceptible level of noisy galvanic vestibular stimulation (nGVS) can improve postural stability in patients with bilateral vestibulopathy during the stimulus, presumably by enhancing vestibular information processing. In this study, we investigated the after-effects of an imperceptible long-duration nGVS on body balance in elderly adults. Thirty elderly participants underwent two nGVS sessions in a randomised order. In Session 1, participants received nGVS for 30 min twice with a 4-h interval. In Session 2, participants received nGVS for 3 h. Two-legged stance tasks were performed with eyes closed while participants stood on a foam rubber surface, with and without nGVS, and parameters related to postural stability were measured using posturography. In both sessions, the postural stability was markedly improved for more than 2 h after the cessation of the stimulus and tended to decrease thereafter. The second stimulation in Session 1 caused a moderate additional improvement in body balance and promoted the sustainability of the improvement. These results suggest that nGVS can lead to a postural stability improvement in elderly adults that lasts for several hours after the cessation of the stimulus, probably via vestibular neuroplasticity.

The vestibular system, which is involved in the detection of linear and angular movements of the head, provides peripheral sensory input in the postural control system and contributes to stabilisation of body balance^1^. Vestibular dysfunction results in dizziness and decreased postural stability, which is prevalent in the elderly^2^.

Galvanic vestibular stimulation (GVS) transcutaneously delivers electrical current to vestibular hair cells and their afferents through electrodes placed over the mastoids^3^,^4^. It has been used to evaluate vestibular function by examining the influence of the electrically stimulated vestibular nerve on postural control^4^. An imperceptible level of GVS delivered as zero-mean current noise (noisy GVS, nGVS) has also been shown to improve baroreflex function in healthy individuals and autonomic and motor functions in patients with neurodegenerative diseases^5^,^6^,^7^. Recently, our group showed that a 30-s application of nGVS can improve postural stability in healthy individuals and in patients with untreatable persistent unsteadiness caused by bilateral vestibulopathy (BV)^8^. This kind of vestibular stimulation also improves dynamic stability during walking in healthy subjects and BV patients^9^,^10^,^11^. The rationale behind these ameliorating effects is stochastic resonance, in which an optimal amount of noise added to nonlinear systems can enhance information processing within the system^12^.

The vestibular system exhibits strong neuroplasticity^13^,^14^. After acute unilateral vestibular dysfunction, vestibular symptoms, such as vertigo and unsteadiness, and abnormal neurological findings, such as spontaneous nystagmus and postural instability, disappear over time, despite incomplete recovery of the damaged vestibular function. This process, known as vestibular compensation, has been attributed to the neuroplasticity of the central vestibular pathways^15^,^16^,^17^. Behavioural studies have shown that the vestibulo-ocular reflex, which elicits compensatory eye movements to keep the retinal image stable during head motion, exhibits powerful motor learning in response to both visual and vestibular stimulation in primates^18^. Physiological experiments have shown that stimulation of the vestibular nerve can induce both long-term potentiation (LTP) and long-term depression (LTD) of the field potentials in the vestibular nuclei in rodents^19^.

Given the strong neuroplasticity of the vestibular system, we hypothesised that nGVS would induce a sustained improvement in postural stability, even after the cessation of the stimulus. The purpose of this study was to determine whether nGVS has a post-stimulation effect on the improvement in body balance in elderly adults. We also examined the cumulative effect of nGVS on the improvement in postural stability using repeated application of nGVS. We revealed that nGVS has both post-stimulation and cumulative effects on improving postural stability in elderly adults that last for several hours. These participants obtained postural stabilisation without stimulation due to persistence of the post-stimulation effect.

## Results

### Determination of the optimal intensity of nGVS for postural stability

**Figure 1:**
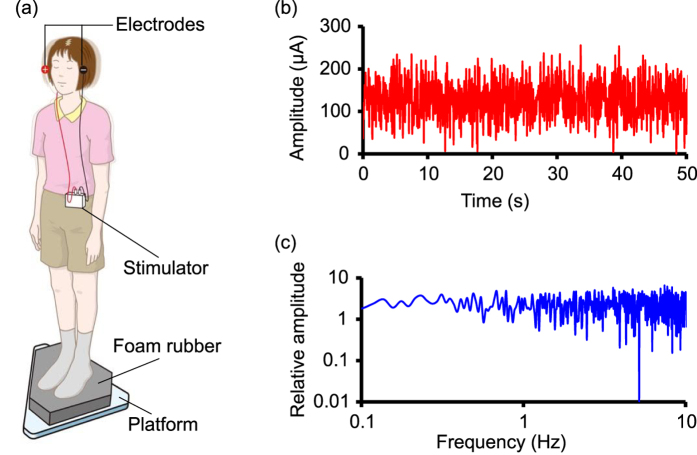
Experimental setup. (**a**) Noisy galvanic vestibular stimulation (nGVS) was applied with electrodes on the right and left mastoids by a portable stimulator. Two-legged stance tasks were performed with eyes closed while participants stood on foam rubber with and without nGVS. **(b)** Waveforms of nGVS are shown. **(c)** A frequency spectrum of nGVS is shown.

**Figure 2:**
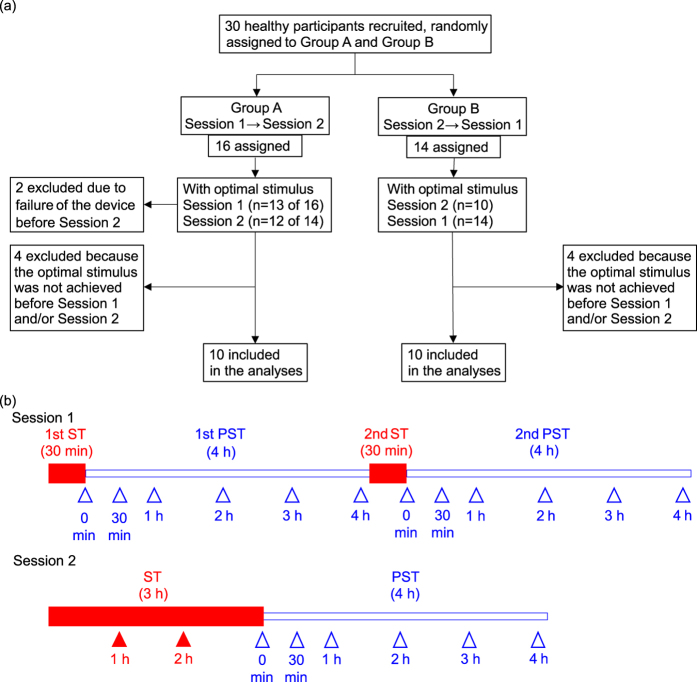
Consolidated Standards of Reporting Trials diagram for the flow of participants through the study and protocols. **(a)** We recruited 30 healthy elderly participants. Among them, 20 participants who had the optimal stimulus before both sessions were finally included in the analyses. **(b)** Two experimental sessions were performed in a randomised order. Arrowheads indicate the measurement time point of posturography. ST = stimulation period, PST = post-stimulation period.

To examine the post-stimulation effect and the cumulative effect of nGVS, which was applied with electrodes on the right and left mastoids (Fig. 1a–c), on postural stability, we performed two experimental sessions (Fig. 2a,b). In Session 1, participants received nGVS for 30 min twice with a 4-h interval (Fig. 2b). In Session 2, participants received nGVS for 3 h. Postural stability was measured using posturography for 4 h after each stimulation (Figs 1a and 2b; see Methods). We measured the following three parameters: the mean velocity (velocity), the envelopment area (area), and the root mean square (RMS) of the centre of pressure (COP) movement in the XY plane. We determined the optimal intensity of nGVS, which improved all of these parameters simultaneously during the stimulus compared with baseline, before each experimental session^8^.

Thirty elderly healthy participants were randomly assigned to first undergo Session 1 and then Session 2 (Group A) or to first undergo Session 2 and then Session 1 (Group B) (Fig. 2a). Two participants were excluded because of mechanical trouble during the examination of the optimal intensity. Overall, in 84% of the sessions (49 of 58 sessions), participants had the optimal intensity of nGVS. The mean intensity of the optimal stimulus was 178.8 (±9.1) μA. No participant experienced an adverse event or felt any sensations in response to these stimuli. Of the 28 participants who completed the examination of the optimal intensity before the two experimental sessions, 20 participants who had the optimal intensity before both sessions (71%; 12 men and 8 women; age range 64–70 years, mean age 66.7 ± 0.4 years) were ultimately included in the analysis (Fig. 2a; see Supplementary Table S1 for baseline characteristics of each session). The optimal intensity of nGVS was used for the subsequent experimental session.

### Post-stimulation effect of nGVS on the improvement in postural stability

**Figure 3:**
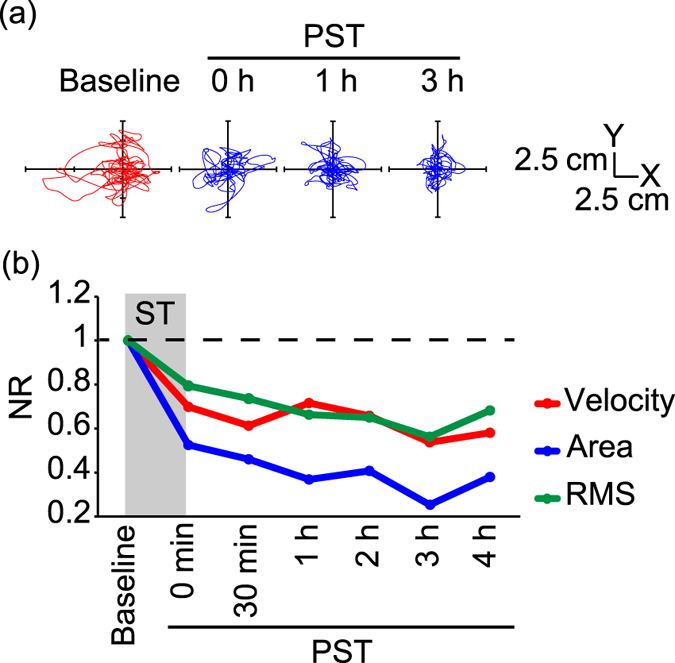
Statokinesigrams and changes in the normalised ratios (NRs) of the velocity, area, and RMS for a representative 67-year-old male participant in the first nGVS session (Session 1). **(a)** Statokinesigrams are shown. **(b)** nGVS for 30 min improved the NRs of all three parameters in the PST. Dashed line indicates NR = 1.0.

We defined the normalised ratio (NR) as the ratio of the value of each parameter at the measurement time point to that at baseline. A representative participant who received nGVS at its optimal intensity for 30 min showed improvements in the NRs of all three parameters of COP just after the cessation of the stimulus (Fig. 3). The improvement reached a peak at a post-stimulation period (PST) of 3 h and continued for more than 4 h.

**Figure 4:**
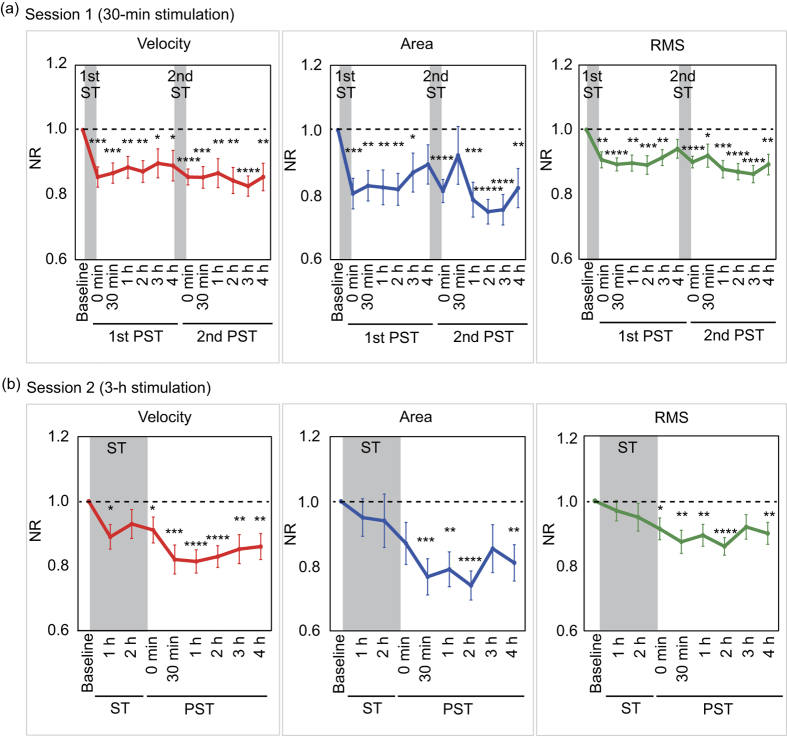
Mean NRs at the measurement time points in Session 1 and Session 2. **(a)** Mean NRs of the velocity, area, and RMS in Session 1 are shown. **(b)** Mean NRs of the velocity, area, and RMS in Session 2 are shown. Dashed line indicates NR = 1.0. NR = normalised ratio, RMS = root mean square, ST = stimulation period, PST = post-stimulation period. *P < 0.05, **P < 0.01, ***P < 0.001, ****P < 0.0001, *****P < 0.00001.

Overall, in Session 1, the NRs of the velocity, area, and RMS were significantly smaller at the immediate PST (0 h) than at baseline (P < 0.01; Fig. 4a). This ameliorating effect continued after the cessation of the stimulus; the NRs of the three parameters at PST 2 h were maintained at almost the same levels as those at PST 0 h. This effect of nGVS began to decrease thereafter, but the NRs of all three parameters were still significantly smaller at PST 3 h than at baseline (P < 0.05).

In Session 2, the effect of nGVS on postural stability during the 3-h stimulation was moderate, but the post-stimulation effect of nGVS on the improvement in postural stability was also observed in this session (Fig. 4b). The NRs of the velocity and RMS were significantly smaller at PST 0 h than at baseline (P < 0.05). The post-stimulation effect of nGVS increased until PST 2 h. The NRs of the velocity, area, and RMS were significantly smaller at PST 2 h than at baseline (P < 0.0001). This effect began to decrease thereafter, but at PST 4 h, the NRs of all three parameters were still significantly smaller than those at baseline (P < 0.01). At PST 4 h, no significant differences were observed in any of the NRs between Sessions 1 and 2 (P > 0.05), but the longitudinal change in the NR of the velocity was significantly smaller in Session 2 than in Session 1 (P < 0.001; see Supplementary Table S2 and Supplementary Table S3).

### Cumulative effect of nGVS on the improvement in body balance

The second 30-min nGVS in Session 1 improved postural stability relative to that just before the stimulus (Fig. 4a). This ameliorating effect of the second single stimulus on the NRs of the area and RMS decreased at PST 30 min, but increased and continued after PST 1 h. The NRs of all three parameters at PST 4 h in the second stimulus were significantly smaller than those at baseline (P < 0.01; Fig. 4a). The longitudinal changes in the NRs of the area and RMS were significantly smaller in the second PST than in the first PST (P < 0.05) (see Supplementary Table S4). These analyses revealed that the second stimulation caused a moderate additional improvement in postural stability and promoted the sustainability of the improvement.

## Discussion

In the present study, we show that nGVS has a significant post-stimulation effect on the postural stability improvement that lasts for several hours in healthy elderly adults. The repetitive application of nGVS for 30 min conferred a moderate additional improvement in postural stability and promoted the sustainability of the improvement.

We have previously shown that nGVS can improve postural stability during the stimulation in healthy volunteers as well as in patients with BV^8^. The mechanism hypothesised to underlie this ameliorating effect is stochastic resonance, in which an optimal amount of noise added to a nonlinear system can enhance the information processing within this system, whereas a further increase in noise intensity degrades information transfer^5^,^6^,^7^,^8^,^20^,^21^,^22^. An appropriate intensity of noise has been shown to intensify the detection of subthreshold signals in various sensory functions such as auditory perception, visual perception, and tactile sensations^23^,^24^,^25^. In the present study, we found an optimal intensity of nGVS in more than 80% of the participants, which simultaneously improved the three COP parameters during the stimulation in most of the sessions, confirming our previous observations^8^.

Here we showed that nGVS has strong post-stimulation effects for improving postural stability. This effect is a novel phenomenon and the mechanism underlying this phenomenon might be vestibular neuroplasticity because the vestibular system shows extensive neuroplasticity^16^. Behavioural analyses have shown that powerful forms of motor learning occur in the vestibulo-ocular reflex, resulting in adaptive changes in the strength and timing of eye movements^18^. It has been shown that this vestibular neuroplasticity resides in synaptic plasticity in the vestibular nuclei as well as in the cerebellar circuit. Experimental studies indicated that learning in the vestibular system initially depends on the activity of Purkinje cells in the cerebellar flocculus, whereas consolidated memories appear to be stored in the vestibular nucleus^19^. High-frequency stimulation of the vestibular nerve can induce LTP and LTD of the field potentials in vestibular nuclei in rodents^19^. The vestibular nuclei receive input from the vestibular end-organs and produce excitation of the ipsilateral extensor motor neurons of the limb and inhibition of reciprocal flexor motor neurons via the lateral vestibulospinal tract^26^. The gain and spatiotemporal properties of the vestibulospinal reflexes are controlled by the activity of the cerebellum^27^. In the present study, white noise GVS might induce synaptic plasticity in the vestibular nucleus, the cerebellum, resulting in the post-stimulation effects of improved postural stability.

The post-stimulation effect of 3-h nGVS was significantly greater than that of 30 min nGVS in terms of the longitudinal change in the velocity, but the difference was relatively small, suggesting that nGVS for 30 min is sufficient to induce a long-term post-stimulation change in postural stability. The strength of neuroplastic changes in the vestibular nuclei is altered dramatically by various factors, such as age and the modalities of the stimulation and visual and vestibular experience^13^,^28^,^29^. Further investigation is needed to clarify the factors determining the appropriate duration of the stimulus to induce long-term post-stimulation effects of nGVS on postural stability.

On the other hand, nGVS for 3 h had little effect on improving postural stability during the stimulation. This might be due to an adaptation of neural systems via rebalancing of the modulation of ion channel conductance after the initial increase in excitability^30^. The effects of nGVS are probably based on increased activation of the voltage-gated ion channels in the vestibular systems. Constant activation by a voltage change causes some ion channels to undergo a progressive decrease in activation. This inactivation of voltage-dependent ion channels may play a key role in determining whether there is a progressive decrease in postural stability in response to sustained exposure to longer stimulation.

Moderate cumulative effects of the 30-min nGVS in Session 1were observed. A previous study of the effects of transcranial direct current stimulation (tDCS), in which low-intensity direct electrical currents are applied to the skull for several minutes, on human motor cortical excitability revealed that repeated cathodal tDCS during the post-stimulation effects of the first stimulus prolonged the duration of the post-stimulation effects, whereas repeated tDCS after the disappearance of the post-stimulation effects of the first stimulus did not have such effects^31^. The study indicated that the regulation of plasticity depends on the timing of stimulation in the motor cortex. The optimisation of the interval between the first and the second nGVS sessions might increase the cumulative effect of nGVS.

BV is a rare disorder that accounts for approximately 4% of vestibular disorders, with an increased frequency in the elderly^32^. The most common causes of BV are ototoxic aminoglycosides, Meniere’s disease, and meningitis. However, the cause of BV is unclear in about half of patients^32^, and it may include inherited, autoimmune, and metabolic causes^33^. Patients with BV have severe clinical symptoms such as persistent unsteadiness, particularly in darkness or on uneven ground, and oscillopsia during head movements and locomotion^32^, but there are no effective treatments for the disease. Vestibular implantation, which directly stimulates the peripheral vestibular nerve through electrical pulses, has been proposed as a therapy for BV^34^,^35^,^36^, but the surgery carries potential risks, such as facial nerve palsy and hearing loss. On the other hand, nGVS is safe, minimally invasive, and without any adverse events. In our previous study, nGVS had ameliorating effects on postural stability in healthy volunteers as well as in patients with BV during short-term stimulation, and the proportion of participants who had the optimal intensity was higher for patients with vestibulopathy than for healthy volunteers^8^. The long-term effects of nGVS, shown in this study, are advantageous for increasing its applicability for improving postural stability in daily life. nGVS could be a promising treatment option for BV if it also has a long-term post-stimulation effect in patients with BV.

This study has several limitations. First, the study was not placebo-controlled. It is possible that habituation of the participants due to the repeated posturography measurements affected the results of the post-stimulation effect of nGVS on postural stability. However, the improvement in the NRs of each parameter decreased after PST 2 h in both the 30-min and 3-h nGVS sessions. These results cannot be explained by the habituation effect and suggest the existence of the post-stimulation effect of nGVS and its attenuation. The second limitation is that we did not continue measuring the postural stability until the post-stimulation effect had completely disappeared. We showed that the NRs of all three parameters were improved for at least 3 h after 30-min nGVS and for at least for 2 h after 3-h nGVS, but the post-stimulation effects of nGVS might continue for more than 4 h.

In conclusion, we revealed that nGVS can induce a long-term improvement in postural stability after the cessation of the stimulus due to a strong post-stimulation effect and that repetition of the stimulus can induce further improvement. These newly discovered effects could contribute to an increased applicability of nGVS for postural stabilisation in elderly adults.

## Methods

### Standard protocol approvals, registrations, and participant consents

All procedures were conducted in accordance with the Helsinki Declaration and were approved by the Institutional Review Board of the University of Tokyo Hospital (P2114052-11Y). This trial was registered with the University Hospital Medical Information Network (UMIN) Clinical Trials Registry (UMIN-CTR: UMIN000016054; date of registration: December 25, 2014). The date defining the periods of recruitment and follow-up was December 24, 2014. All participants gave written informed consent.

### Participants

Thirty healthy participants (17 men and 13 women; age range 64–70 years, mean age 67.0 [±0.3] years) were recruited from a human resource centre (Fig. 2a,b). Exclusion criteria included episodes of vertigo/dizziness, hearing loss except presbycusis, ear diseases, orthopaedic diseases, cardiovascular diseases, malignant tumour, psychiatric diseases, use of tranquillisers or antidepressants, consumption of alcohol after 10pm on the evening before testing, presence of metal objects in the body, and inability to walk without assistance.

### Procedures

To examine the post-stimulation and cumulative effects of nGVS on postural stability, we performed two experimental sessions, separated by an interval of 7 days, after determining the optimal intensity of nGVS (Fig. 2b). In Session 1, participants received nGVS at its optimal intensity for 30 min twice with a 4-h interval; postural stability was measured for 4 h after each nGVS. In Session 2, participants received nGVS at its optimal intensity for 3 h and were monitored without stimuli for 4 h; postural stability was measured during and after the nGVS. During and after the stimulation, participants had no restrictions in their activities and were allowed to act freely in the hospital. The order of the sessions was randomly determined (Fig. 2a). The participants were randomly assigned through the use of a computer-generated permuted-block design with block size 2, in a 1:1 ratio, to first undergo Session 1 and then Session 2 (Group A) or to first undergo Session 2 and then Session 1 (Group B). The Clinical Research Support Center at the University of Tokyo Hospital generated the random allocation sequence, and an independent academic biostatistician performed all analyses for this article. Enrolment was performed by the investigators.

### Posturography

Instantaneous fluctuations in the position of the COP were determined by using a Gravicorder GP-5000 (Anima Co. Ltd., Tokyo, Japan; sampling frequency, 20 Hz) with a foam rubber surface containing vertical force transducers. The data were plotted in a statokinesigram (i.e., the sway path of the COP). The foam material consisted of natural rubber (tensile strength, 2.1 kgf/cm^2^; stretch percentage, 110%; thickness, 3.5 cm). Two-legged stance tasks were performed by healthy participants with eyes closed while participants stood on the foam rubber surface, with and without nGVS (Fig. 1a). Three parameters were measured in the XY plane: the mean velocity of the movement of the COP (velocity), the envelopment area traced by the movement of the COP (area), and the RMS of the COP distance^8^.

### nGVS

nGVS was applied with electrodes on the right and left mastoids by a portable stimulator (112 × 67 × 28 mm; 200 g including dry cells) (Fig. 1a)^7^,^8^. GVS waveforms were stored digitally and converted from digital to analogue at 20 Hz. We used white noise GVS, which ranged from 0.02 to 10 Hz (Fig. 1b,c).

We determined the optimal intensity of nGVS for each participant before initiating each experimental session investigating the long-term effects. First, the value of each COP parameter with no stimulation (0 mA) for 30s was measured and defined as the baseline value. Then, nGVS was applied for 30s with peak amplitudes set at 50, 100, 200, 300, and 500 μA. The optimal intensity was defined as the one at which the value measured during the stimulation was simultaneously smaller than that at baseline in all of the three COP parameters^8^.

### Outcome measures

In Session 1, posturographic data were analysed at 0, 0.5, 1, 2, 3, and 4 h during the PST (Fig. 2b). In Session 2, posturographic data were analysed at 1 and 2 h during the stimulation period (ST) and at 0, 0.5, 1, 2, 3, and 4 h during the PST (Fig. 2b).

We defined the normalised ratio (NR) as the ratio of the value of each parameter at the measurement time point to that at baseline. The primary endpoint was defined as the NRs of the velocity, area, and RMS at PST 4 h in the first part of Session 1 and Session 2. Secondary endpoints were defined as follows: (1) the NRs of the velocity, area, and RMS at PST 0 h in the first part of Session 1 and Session 2; (2) amount of change from baseline in the NRs of the velocity, area, and RMS in the first part of Session 1 and Session 2; (3) longitudinal change in the NRs of the velocity, area, and RMS in the first PST of Session 1 and the PST of Session 2; and (4) longitudinal change in the NRs of the velocity, area, and RMS in the first and second PST in Session 1.

### Data analysis

Data are expressed as mean ± standard error. Statistical analysis was performed using SAS software version 9.4 (SAS Inc., Cary, NC, USA). A paired t-test was performed for the comparison of the NRs of the velocity, area, and RMS at a certain measurement time point during the PST between the first part of Session 1 and Session 2 and between the first and second parts of Session 1. Generalised linear mixed models were adopted for the comparison of longitudinal changes in the NRs of the velocity, area, and RMS between the first PST of Session 1 and the PST of Session 2. Generalised linear mixed models were also used for the comparison of the longitudinal change in the NRs of the velocity, area, and RMS between the first PST and second PST in Session 1. A one-sample t-test was performed to assess the change in the NRs of the velocity, area, and RMS from baseline. P < 0.05 was considered statistically significant.

## Additional Information

**How to cite this article**: Fujimoto, C. *et al*. Noisy galvanic vestibular stimulation induces a sustained improvement in body balance in elderly adults. *Sci. Rep*. **6**, 37575; doi: 10.1038/srep37575 (2016).

**Publisher’s note**: Springer Nature remains neutral with regard to jurisdictional claims in published maps and institutional affiliations.

## Supplementary Material

Supplementary Information
