# Supplementary material for: Noisy galvanic vestibular stimulation induces a sustained improvement in body balance in elderly adults
Source: Sci Rep. 2016 Nov 21;6:37575. doi: 10.1038/srep37575 (PMC5116631; doi:10.1038/srep37575)
Supplement: Supplementary Information [file srep37575-s1.pdf]

Noisy galvanic vestibular stimulation induces a sustained improvement in body balance in elderly adults

Chisato Fujimoto[1], Yoshiharu Yamamoto[2], Teru Kamogashira[1], Makoto Kinoshita[1], Naoya Egami[1], Yukari Uemura[3], Fumiharu Togo[2], Tatsuya Yamasoba[1] and *Shinichi Iwasaki[1]

1 Department of Otolaryngology, Faculty of Medicine, The University of Tokyo

2 Educational Physiology Laboratory, Graduate School of Education, The University of Tokyo

3 Biostatistics Division, Clinical Research Support Center, The University of Tokyo Hospital

Corresponding author: Shinichi Iwasaki MD, PhD (iwashin-tky@umin.ac.jp)

Department of Otolaryngology, Faculty of Medicine, The University of Tokyo, 7-3-1, Hongo, Bunkyo-ku, Tokyo, Japan

Telephone number: +81-3-5800-8665, Fax number: +81-3-3814-9486,

Co-authors: Chisato Fujimoto MD, PhD (cfujijmoto-tky@umin.ac.jp), Yoshiharu Yamamoto PhD (yamamoto@p.u-tokyo.ac.jp), Teru Kamogashira MD (tkamogashira-tky@umin.ac.jp), Makoto Kinoshita MD, PhD (kinoshitam-zao@umin.ac.jp), Naoya

Egami MD (n-egami@umin.ac.jp), Yukari Uemura PhD (yukariuemura-tky@umin.ac.jp), Fumiharu Togo PhD (tougou@p.u-tokyo.ac.jp), Tatsuya Yamasoba MD, PhD (tyamasoba-tky@umin.ac.jp)

Statistical analysis by Yukari Uemura PhD, Biostatistics Division, Clinical Research Support Center, The University of Tokyo Hospital

Supplementary Table S1 Baseline characteristics of each session for finally included participants.

		Session 1	Session 2
Number of participants		20	20
Age (years)	Mean ± SE	66.7 ± 0.4	66.7 ± 0.4
	Minimum value	64	64
	Median	66.5	66.5
	Maximum value	70	70
Body mass index (kg/m^2)	Mean ± SE	22.8 ± 0.5	22.8 ± 0.5
	Minimum value	19.1	19.1
	Median	22.3	22.3
	Maximum value	28.4	28.4
Velocity (cm/s)	Mean ± SE	3.54 ± 0.20	3.79 ± 0.26
	Minimum value	2.27	2.36
	Median	3.32	3.67
	Maximum value	5.75	6.77
Area (cm^2)	Mean ± SE	17.99 ± 1.51	18.87 ± 1.47
	Minimum value	8.26	10.64
	Median	16.72	16.57
	Maximum value	35.95	32.40
RMS (cm)	Mean ± SE	1.77 ± 0.07	1.80 ± 0.07
	Minimum value	1.19	1.36
	Median	1.76	1.76
	Maximum value	2.44	2.42

SE = standard error. RMS = root mean square.

Supplementary Table S2 Comparison of NR at PST 4 h in Session 1 and NR at PST 4 h in Session 2.

		Average (SE)		P value
		Session 1	Session 2	
NR	Velocity	0.89 (0.05)	0.86 (0.04)	0.63
	Area	0.89 (0.06)	0.81 (0.06)	0.30
	RMS	0.94 (0.03)	0.90 (0.03)	0.35

PST= post-stimulation period, NR = normalized ratio, SE = standard error, RMS = root mean square

Supplementary Table S3 Generalized linear mixed models to compare longitudinal change of NR of velocity, area and RMS between the first PST of Session 1 and the PST of Session 2.

			Velocity			Area			RMS		
Factors	Levels		Estimate	SE	P Value	Estimate	SE	P Value	Estimate	SE	P Value
Intercept			1.019	0.031	<.0001	1.007	0.049	<.0001	1.005	0.024	<.0001
Measurement time point	Baseline		0	-	-	0	-	-	0	-	-
	PST	0 min	-0.125	0.021	<.0001	-0.133	0.040	0.001	-0.073	0.020	0.0004
		30 min	-0.145	0.021	<.0001	-0.134	0.040	0.0009	-0.082	0.020	<.0001
		1h	-0.144	0.022	<.0001	-0.177	0.040	<.0001	-0.092	0.020	<.0001
		2 h	-0.133	0.021	<.0001	-0.165	0.040	<.0001	-0.086	0.020	<.0001
		3 h	-0.13	0.021	<.0001	-0.127	0.040	0.002	-0.072	0.020	0.0004
		4 h	-0.119	0.021	<.0001	-0.124	0.040	0.002	-0.067	0.020	0.001
Session	Session 1 (Stimuli for 30min)		0	-	-	0	-	-	0	-	-
	Session 2 (Stimuli for 3h)		-0.041	0.011	0.0003	-0.029	0.021	0.164	-0.016	0.011	0.131
Assigned group	Group A		0	-	-	0	-	-	0	-	-
	Group B		0.0045	0.039	0.908	0.014	0.056	0.808	0.007	0.027	0.791

PST = post-stimulation period, NR = normalized ratio, RMS = root mean square, SE = standard error

Supplementary Table S4 Generalized linear mixed models to compare longitudinal change of NR of velocity, area and RMS between the first PST and the second PST in Session 1.

			Velocity			Area			RMS		
Factors	Levels		Estimate	SE	P Value	Estimate	SE	P Value	Estimate	SE	P Value
Intercept			0.863	0.038	<.0001	0.818	0.058	<.0001	0.909	0.028	<.0001
Measurement time point	PST	Baseline	0	-	-	0	-	-	0	-	-
		30 min	0.009	0.016	0.569	0.048	0.033	0.142	0.007	0.016	0.638
		1h	0.018	0.016	0.285	-0.011	0.033	0.748	-0.011	0.016	0.506
		2 h	0.022	0.016	0.172	0.005	0.033	0.875	0.00008	0.016	0.996
		3 h	0.021	0.016	0.203	0.026	0.033	0.436	0.004	0.016	0.7978
		4 h	0.038	0.016	0.020	0.041	0.033	0.210	0.018	0.016	0.261
Session	First stimulation in Session 1		0	-	-	0	-	-	0	-	-
	Second stimulation in Session 1		-0.018	0.009	0.0525	-0.041	0.019	0.031	-0.024	0.009	0.0089
Assigned group	Group A		0	-	-	0	-	-	0	-	-
	Group B		0.00399	0.054	0.463	0.122	0.078	0.131	0.066	0.038	0.094

PST = post-stimulation period, NR = normalized ratio, RMS = root mean square, SE = standard error
